# Supplementary material for: Predictive modeling of consumer purchase behavior on social media: Integrating theory of planned behavior and machine learning for actionable insights
Source: PLoS One. 2023 Dec 27;18(12):e0296336. doi: 10.1371/journal.pone.0296336 (PMC10752534; doi:10.1371/journal.pone.0296336)
Supplement: Attachment — Submitted filename: Response to Reviewers.pdf [file pone.0296336.s001.pdf]

September 26, 2023

Editor-in-Chief
PLOS ONE

Dear Editor-In-Chief,

We want to express our sincere gratitude for granting us the chance to submit the revised manuscript (Ref. No.: PONE-D-23-21252) titled "Predictive modeling of consumer purchase behavior on social media: integrating theory of planned behavior and machine learning for actionable insights" to PLOS ONE. We greatly value the time and effort you and the reviewer invested in our manuscript, which has significantly improved it.

Your feedback and the reviewer's suggestions were truly invaluable. We are pleased to inform you that we have carefully addressed all the reviewer's questions and comments, making the necessary revisions to the manuscript.

Our responses to the reviewer's comments are included in the following pages, with page numbers referring to the revised manuscript that incorporates tracked changes. As the corresponding author, I confirm that all authors have reviewed the manuscript, agreed to the changes, and consented to the submission of this revised version.

We genuinely appreciate your consideration of our manuscript.
Best Regards,

Dr. Sifat Momen (Corresponding Author)
Associate Professor
Department of Electrical and Computer Engineering
North South University, Dhaka, Bangladesh
E-mail: sifat.momen@northsouth.edu

Response to The Editorial Board

Academic Editor's Comments

1. Please ensure that your manuscript meets PLOS ONE's style requirements, including those for file naming.

   Response:

   We have ensured that the manuscript maintains the PLOS ONE style requirements.

2. Please note that PLOS ONE has specific guidelines on code sharing for submissions in which author-generated code underpins the findings in the manuscript. In these cases, all author-generated code must be made available without restrictions upon publication of the work. Please review our guidelines at https://journals.plos.org/plosone/s/materials-and-software-sharingloc-sharing-code and ensure that your code is shared in a way that follows best practice and facilitates reproducibility and reuse.

   Response:

   Thank you for pointing this out. Our works and findings have now been shared via a GitHub repository. You can find it at `https://github.com/shawmoonazad/Purchase_Behavior_TPB`. It also includes necessary documentation, running instructions and the output results that underpin the findings of the paper.

Note to The Reviewers

1. All modified portions in the revised manuscript are described in the blue-colored text.
2. In answering the comments, we have used the following format throughout this response letter. We first state the comments made by the respective reviewer, followed by our response.

[Tribute] We would like to express our deepest appreciation to the anonymous reviewer for extremely insightful comments that greatly enhanced the quality of the paper. We have made every effort to implement the suggestions demonstrating the effectiveness of our work.

Response to The Reviewers

Responses to the review comments are illustrated below:

Reviewer 1

Comment 1.1

Abstract is structured well but it only talks about the findings in the way it is represented in the paper. It would be better if the abstract explains the outcome with implications.

Response:

First of all, we would like to sincerely appreciate the time you have taken to provide such a constructive and detailed review of our work. Based on your remark, we have now included the implications of the findings in the abstract. The modifications can be found at the end of the abstract.

Comment 1.2

Introduction should also address: Why the current research is required? How the research contributes to the existing body of knowledge?

Response:

Thank you for this insightful comment. In response to the query regarding the necessity of this research, we have thoughtfully incorporated a rationale into the introduction, specifically within lines 33 to 44 of the revised manuscript. This addition explains why our current research holds significance in the broader context. The contribution of our research is indicated in lines 45 – 48. Furthermore, we have summarized our key contributions in lines 49 – 62. Moreover, we have probed the existing literature and identified research gaps, as evidenced in lines 172 to 187. Our research work operates within these gaps and contribute in filling a void in the current knowledge

landscape. This highlights the uniqueness of our contribution and emphasizes the importance of our research within the broader research community.

Comment 1.3

Literature needs to be strengthened from TPB perspective. Why and How TPB is suitable for this study and how it has been used in past?

Response:

Thank you for your comment. We have rewritten the whole subsection related to TPB perspectives. We believe that the literature has been strengthened from TPB angle. The revision can be found in lines 72 to 118 (pages 3 and 4). Theory of planned behavior is a widely recognized social psychological theory that investigates and explains human behavior, particularly in the context of decision-making and intention formation. Machine learning also looks into predicting outcomes. Explainable AI looks into explaining the predicted outcomes The two domains (TPB and Machine learning with Explainable AI) have many common goals but they have not been integrated well in the literature. This has been a source of motivation for us to integrate the two domains as discussed throughout the paper.

Comment 1.4

A strong recommendation is to highlight the literature gap.

Response:

Thank you for bringing this to our attention, and we greatly appreciate your feedback. We have now included a new subsection (lines 172 – 187) addressing the research gap in past literature.

Comment 1.5

Research methodology should address the following issues:
Clarity on study design
Sampling design
Area of study, which state/ region?
Method of data collection and why?
Common method bias

Non-response bias
Demographic profile of respondent (Table)

Response:

As suggested, we have updated the research methodology accordingly. To address this, we have rewritten the research methodology section from page 6 to page 10, providing more clarity on the study design. We have also addressed the sampling design, the area of study, and the method of data collection (and its reasonings) in the data acquisition subsection on pages 7 and 8 (lines 201 to 233). Please note that the dataset collected pertains to Malaysia; however, the authors [15] who collected the dataset have not specifed any specific state or region within Malaysia from which the dataset originates. We have also addressed common method bias and non-response bias issues in the manuscript, which can be found on pages 9 and 10 (lines 236 to 269). Furthermore, we have included a table presenting the demographic profile of the 219 respondents on page 8.

Comment 1.6

Data visualization can be done in a better way in terms of placement of charts/ figures.

Response:

Thank you for your feedback. We have looked into this issue and took necessary steps to resolve it. We believe that the charts/figures now have improved placements.

Comment 1.7

A complete sub-section required for following:
Managerial Implication
Social Implication
Future research directions

Response:

Thank you for this valuable suggestion. To address it, we have introduced three new subsections in the manuscript. Specifically, we have included subsections "Managerial Implications" and "Social Implications"

under the “Research Implications” section on page 25. Additionally, we have added a “Future Research Directions” subsection on page 26 (lines 577 to 585).
